# Measuring and evaluating the influence of cultural sustainability indicators on sustainable cultural tourism development: Scale development and validation

**DOI:** 10.1016/j.heliyon.2025.e42514

**Published:** 2025-02-07

**Authors:** Sadanand Gaonkar, Sitaram V. Sukthankar

**Affiliations:** a Goa Business School, Goa University, Goa, India; b Government College of Arts, Science and Commerce, Khandola, Marcela, Goa, India

**Keywords:** Cultural tourism, Cultural sustainability indicators, Intangible cultural heritage, Scale development, Sustainable cultural tourism development

## Abstract

In the past few decades, tourism has significantly impacted the ecological, social, and environmental dimensions, making sustainability facilitation more vital. These aspects are also regarded as the primary pillars of sustainable tourism development. Culture is the cornerstone for accomplishing sustainable tourism research and practice goals in many domains. Culture has received very little attention despite being the deeply rooted and reliable framework for human existence. As a part of cultural tourism, even Intangible Cultural Heritage (ICH) has been largely ignored in sustainable tourism development. Moreover, the varied sustainability dimensions do not explain cultural sustainability indicators and how these indicators influence sustainable cultural tourism development. Therefore, firstly, this research aims to develop and measure a new scale for Cultural Sustainability Indicators and Sustainable Cultural Tourism Development (SCTD) and, secondly, to examine the influence of Cultural Sustainability Indicators on SCTD growth. In this sense, data was gathered from tourism stakeholders using self-administered questionnaires. A total of 1000 respondents completed the questionnaire; 949 of those responses were deemed legitimate. The new scale development identified the seven most important cultural sustainability indicators— Authenticity, Awareness, Commodification and Transformation, Empowerment, Parallel Development, Promotion, and Sustainability Practices, and one indicator of SCTD growth—SCTD, through Exploratory Factor Analysis (EFA) in SPSS 20. Further, to investigate the influence of cultural sustainability indicators on the growth of SCTD, additional analysis is conducted using PLS-SEM in Smart PLS 4. The study results showed that, except for parallel development and promotion, the five cultural sustainability indicators significantly and positively influence SCTD. Thus, the study concludes that culture is one of the most important aspects of developing a sustainable tourism industry and may be used to assess the sustainability of tourist destinations. The primary contribution of this work is a scale that depicts current perspectives and solutions for the cultural tourism sector as perceived by different stakeholders.

## 1 Introduction

Sustainable tourism emerged in the early 90s by connecting sustainable development ideas and principles with tourism. Many international and national organisations and other researchers quickly accepted and promoted this new concept by defining the terms and dimensions depending on their context and perception [1]. However, it is widely accepted and agreed that sustainable tourism is an aspect and contribution to sustainable development. The [2], in “Our Common Future”, defined sustainable development as “the development that meets the needs of the present without compromising the ability of future generations to meet their own needs”. This brought sustainable development further into the political arena and positively affected government and non-government organisations. Activities that meet the current social, economic, and environmental needs without severely impacting the natural and cultural environment so that future generations can provide them equitably accessible [3].

Facilitating sustainability has been imperative in the tourism sector over a few decades due to the impact of tourism activities on the ecological, social, and environmental dimensions [3,4]. Culture is the highest level, the most ingrained and stable frame of reference in human life, and is increasingly recognised as the essential foundation of any spatial analysis [5,6]. Over the last decade, culture has been elevated as the fourth pillar of sustainability alongside economic, social, and ecological sustainability [7]. Several compelling reasons exist to integrate culture into the traditional three-pillar sustainability framework. Culture encompasses a society's beliefs, values, practices, and aspirations, shaping how these values are expressed and applied in everyday life and preserved and transmitted to future generations [8]. Individuals should develop certain values and behaviours to achieve environmental responsibility, social equity, and economic development [9]. Since a sustainable society relies on a sustainable culture, efforts to meet sustainable development goals must address the natural, social, economic and cultural aspects. If a society's culture deteriorates, its other components will unravel [8]. Therefore, ‘culture is fundamental to the possibility of a sustainable society’ [10]. Recognising this, UNESCO's 2001 Universal Declaration on Cultural Diversity initiated the inclusion of culture as the fourth pillar of sustainability [11]. Theoretically and empirically, it has been used to understand how sustainability works and explain its fundamental role in achieving its goals in various scientific and non-scientific disciplines [5,12]. Numerous studies in recent years have shown that tourism may invigorate, enrich, sustain, and further develop destination cultures [[13], [14], [15], [16], [17], [18], [19], [20], [21]]. As a vital resource and asset for any country, culture can contribute to sustainability by providing social, economic, and spatial benefits [22].

Around the world, culture broadly acknowledges sustainable development as a critical indicator for growth and development. Thus, promoting cultural sustainability is one of the essential objectives at this stage, positively impacting ensuring high-quality development, as it will comprehensively maximise the benefits of sustainable cultural tourism development. Regrettably, the academic community has focused on economic, environmental, and social goals, exploring its policies and sustainable development path [23], and rarely considers culture an independent goal. So, targeted recommendations to improve cultural sustainability in any region may need to be revised [24]. Commercial concerns and infrastructure investment are sometimes prioritised over culture [25], requiring heightened attention. Colombia, Florence, and London are developing cultural preservation zones with limited commercialisation to conserve culture, people, and the environment [26].

As a part of cultural tourism, Intangible Cultural Heritage (ICH) is “the practices, representations, expressions as well as the knowledge and skills-as well as the instruments, objects, artefacts, and cultural spaces associated in addition to that, communities, groups and in some cases, individuals recognise as part of their cultural heritage” [27]. ICH provides vital cultural authenticity and offers tourists a deep understanding of a destination's culture. Enhancing the competitiveness of ICH within the broader cultural heritage tourism sector and generating socio-economic benefits for stakeholders. However, the commodification of ICH as a tourism product has threatened its authenticity, which is crucial for the cultural continuity of communities. To mitigate this [28], has recommended approaches that promote ICH as a sustainable tourism resource, emphasising safeguarding its cultural values. This responsibility lies largely on the stakeholders, especially residents, tourists, and tourism organisations, who learn, practice, maintain, or promote ICH and need more decision-making power. Thus, it is important to explore perspectives of the state's rich cultural resources, including festivals, folk dances, folklore, and traditional customs, which present significant opportunities for attracting cultural tourists. Therefore, it is important to note that cultural sustainability is dynamic and takes different cultural groups' local values and practices as its starting point for sustainable living.

For educators, the challenge is to advocate for the revival and preservation of essential traditions and beliefs while adapting, modifying, and promoting diversity in ways that resonate with rural and urban communities. It is argued that an integrated approach is needed to address cultural sustainability issues. Such an integrated approach could help address better management issues related to decision-making and control over the fair distribution, use, and conservation of resources and achieve the desired goals of Sustainable Cultural Tourism Development (SCTD). Therefore, firstly, this study aims to develop a new scale of cultural sustainability indicators and SCTD growth. Secondly, the research examines the influence of cultural sustainability indicators on the growth of SCTD.

A comprehensive analysis of the relevant literature in tourism studies reveals that the rich knowledge domains of sustainable tourism development have been evolving primarily along parallel pathways in tourism. The paper traces some key developments and offers a framework to bridge these disparate discourses and ensure that cultural sustainability in tourism development and management is grounded in the community as a key principle. The focal point of the research work goes directly to the main research question, i.e., what are the main indicators of cultural sustainability and SCTD growth, and how do cultural sustainability indicators affect SCTD?

The paper is structured as follows. (i) The introduction section provides an overview of the research topic, setting the context for the study by discussing the importance of cultural sustainability and SCTD and outlining the study's main research question and objectives. (ii) The approach section describes the systematic review of the research literature on cultural sustainability, SCTD, and their interrelationship, providing a theoretical foundation for the study. (iii) The method section details the steps involved in the scale development process, including the research design, data collection methods, and analysis procedures. (iv) The results and analysis section presents and discusses the study's key findings. (v) A preliminary framework for SCTD is then proposed, followed by critical directions to address the pressing challenges that continue to hinder research and practical applications.

This study contributes valuable insights in raising global awareness about the importance of living more sustainably in the present and the future. The research also contributes to bridging cultural sustainability indicators and SCTD. Practically, the study aims to support the efforts to safeguard and preserve local ICH, foster the growth of cultural industries, and enhance the appeal and reputation of metropolitan areas. These contributions aim to mitigate urban challenges and cultural homogenisation while promoting cultural diversity and sustainable urban development.

## 2 Literature review

### 2.1 Cultural sustainability indicators

The definition and operationalisation of cultural sustainability are challenging, and different authors have different ideas about it [29]. Cultural sustainability was first defined by the World Commission on Culture and Development as “inter- and intra-generational access to culture” [30]. According to Ref. [31], cultural sustainability “refers to the ability of people or a people to keep or adapt components of their culture which separate them from other people”. The concept of cultural sustainability, as a constructive reaction to identity loss, contests the detrimental effects of tourism on acculturation [32]. However, it is only a small portion of a larger section against the excesses of globalisation [33]. The principle underlying cultural sustainability is that the current generation can engage with and modify cultural heritage only to the extent that it does not hinder future generations from fully understanding and experiencing its diverse values and meanings resources [34]. Thus, this dimension of sustainability is primarily concerned with ensuring the continuity of cultural values that link the past, present, and future [35].

The cultural aspects in the literature on sustainable tourism have frequently been discussed over the last few decades. Nonetheless, there has been significant advancement in the comprehension and expression of the effects of sustainability studies. Since the cultural elements are not specifically addressed in the tripartite sustainability model of economic, social, and environmental dimensions, a comparable concern for the influence on cultural inheritance remains a relatively underarticulated perspective [22]. Numerous researchers have examined the dual impacts of tourism on cultural resources and heritage. On the positive side, tourism can significantly enhance the quality of life by generating employment, attracting investments, and improving community services. It plays a crucial role in preserving cultural events, such as festivals, which foster intercultural understanding and community solidarity [36,37], contribute to the preservation of local culture [38,39] and the improvement of local services [40]. Moreover, cultural tourism can stimulate regional economic development by effectively utilising existing resources and tapping into local capabilities [[41], [42], [43], [44], [45]]. It can improve the self-esteem of cultural custodians and provide economic benefits to performers of ICH [46].

Conversely, tourism can have detrimental effects, including the erosion of local culture, increased crime rates, and environmental challenges such as pollution and overcrowding [47,48]. Furthermore, tourism can lead to commodifying cultural practices, sidelining traditional practitioners [49], potentially damaging the authenticity of local heritage, eroding socio-cultural assets, and producing inauthentic cultural products [50]. Moreover, tourism-related ecological impacts in parks and protected areas can negatively affect their cultural ecosystem services, particularly regarding human health and well-being [51].

A case study of Zhouzhuang, by Ref. [52], reveals the risks of commodifying local culture and transforming authentic lifestyles into tourist experiences. The study highlights the delicate balance between tourism growth and cultural identity preservation, advocating for the involvement of local communities and implementing strategic policies to protect Zhouzhuang's cultural integrity. In the Okavango Delta, tourism has contributed to enclave development, racial tensions, the displacement of traditional communities, and the breakdown of family structures [53]. Additionally, changes in land use have been linked to cultural shifts, undermining key indicators of cultural sustainability [54]. The arts, vital to cultural preservation, often face challenges such as elitist dominance and limited integration into public education, largely due to inadequate government funding [55].

The cultural sustainability lens has also been used in other studies to critically examine the setting and artefacts of tourism, such as museum souvenirs [56], Cypriot museums [57], and UNESCO World Heritage Sites [58]. The Peruvian Amazon and Iranian geoparks are two examples of cultural sustainability that have attracted attention in recent years in tourism [59,60]. These authors argue for a model of ecotourism that goes beyond economic and environmental concerns and prioritises “cultural equity and participatory democracy”, ensuring that local traditions and values are respected and sustained over time. [59], have described how ecotourism impacts the Peruvian Amazon's local cultural practices and traditions. The Posada Amazon as eco-lodge case study highlights the challenges the indigenous and local communities face in balancing the preservation of their cultural identity with the pressures of global tourism markets, appealing to “locally defined and culturally embedded relations and meanings” [60]. stated that geoparks are designed not only to protect geological features but also to integrate cultural heritage. This includes supporting local traditions, knowledge systems, and artisanal practices, making cultural sustainability a key objective in geopark management. These geo products, such as trilobite clocks and ammonite bread, are linked to the region's geological features and help preserve cultural practices while generating economic benefits, appealing to the recovery and protection of cultural identities.

To address these challenges and ensure sustainable development, collaboration among stakeholders in policy formulation, implementation, and monitoring is crucial [53]. Furthermore, cultural commissions and traditional councils should actively promote cultural vitality and resolve conflicts that impede eco-cultural sustainability [54]. Managing tourism's effects is essential to safeguard cultural resources while maximising local benefits. The relationship between tourism and cultural preservation is complex, with the potential for both beneficial and adverse outcomes. To mitigate negative impacts, it is vital to adopt responsible management practices that engage local communities, foster cultural education, and embrace balanced development strategies [61].

### 2.2 Sustainable cultural tourism development

More recently, sustainable tourism has also regularly been linked with preserving ecosystems and biodiversity, promoting human welfare and inter- and intra-cultural equity, public participation in tourism-related decision-making, socio-cultural focus, and access by all stakeholders to socio-cultural tourism outcomes [[62], [63], [64]]. Sustainability can be a policy or development goal [65] for most types of tourism activity or environment, regardless of scale [[66], [67], [68]]. Since the early 1990s, sustainable tourism development has represented the dominant discourse in academic circles and, as such, sustainable tourism has come to be widely embraced by the academic community as a broad conceptualisation that embraces environmental issues in conjunction with social, cultural, economic, and political issues [69].

Moreover [70], has provided the six dimensions of sustainable cultural tourism: sites, tourists, sociocultural aspects, historical aspects, economic revenues, and institutional aspects. The study by Ref. [71] mentioned the benefits of micro-medium cultural events that can connect strongly with the destination's vocations through public-private collaboration and establishing a portfolio of recurrent events. While assessing the sustainable Mi'kmaw cultural tourism development in Nova Scotia, Canada [72], it was found that Mi'kmaw people protect the culture as it brings economic opportunities. However, waste disposal problems, crime, loss of authenticity, lack of information, facilities, tourist services [73], lack of local people involvement [74], environmental concerns, climate change impacts, crisis management, and cultural development plans [75] are some of the constraints while preserving and reviving the socio-cultural heritage of its community.

The Triple Bottom Line (TBL) framework has also described the economic, social, and environmental dimensions as sustainable tourism development indicators [76]. A study by Ref. [77] applied the TBL approach to assist the destination in progressing to become a sustainable cultural heritage tourism destination. It revealed that residents had recognised the need to preserve their social and ethical values actively but failed to protect their abundant environmental resources. Further, a blended approach of three theories, i.e., Stakeholder Theory, Social Network Analysis (SNA), and Actor-Network Theory (ANT), has been applied to conceptualise the network in sustainable tourism development [78]. [79] has explored six issues that are often overlooked, such as the role of tourism demand, the nature of tourism resources, the imperative of intra-generational equity, the role of tourism in promoting socio-cultural progress, the measurement of sustainability, and forms of sustainable development.

A dynamic model proposed by Ref. [80] suggests that cultural managers should evaluate the cultural asset's benefits, thereby protecting the investment, crowding, and resident tourism participation ratios, which may lead to sustaining the development of cultural tourism. A normative analysis approach towards sustainable cultural and heritage tourism has positively impacted developing infrastructure, creating opportunities to attract foreign investors and funding agencies, designing and implementing marketing and promotional strategies, and offering diversified products and services in the cultural tourism market [81]. Therefore, sustaining tourism development needs residents' support, participation in tourism planning [82], and practices in MICE tourism [83]. Furthermore [84], three distinct institutional antecedents of sustainable development in cultural heritage tourism have been identified: governance mechanisms, community agency, and the influence of supranational institutions. This highlights the multidimensional nature of sustainable cultural heritage tourism, which operates across institutional and sectoral domains.

In the post-COVID-19 pandemic period in Bangladesh, sustainable tourism development positively correlated with environmental integrity, social equity, economic prosperity, and technological adaptation. However, partnership enhancement did not demonstrate a significant relationship with sustainable tourism growth [85]. Thus, underpinned by sustainability, sustainable tourism aims to meet communities’ economic and social needs while maintaining and transmitting cultural, economic, and social values.

### 2.3 Influence of cultural sustainability indicators on sustainable cultural tourism development

Cultural sustainability is increasingly recognised as a crucial aspect of sustainable tourism development. Cultural heritage tourism can contribute to economic growth while preserving local identities and traditions [86]. However, mass tourism risks cultural and environmental sustainability, potentially commodifying local cultures and damaging ecosystems. An eco-cultural justice framework has been proposed to address these challenges, emphasising equity and well-being for diverse groups and their cultural heritage [87]. SCTD requires balancing economic benefits with cultural preservation and environmental protection for long-term gain [35,88]. Community involvement, resident access, host connections to tourists, crime and harassment, cultural promotions, ownership patterns, cultural preservations, learning, and authenticity are among the mixed approaches of social and cultural sustainability indicators [89].

Scholars increasingly include sustainability as a theme in their cross-cultural study because of its growing importance. Cultural differences exist in consumers' attitudes, practices, and understanding of “sustainable” [90]. Culture influences consumers' perceptions of sustainability in various circumstances and their assessment of sustainability projects [90,91]. Variations in sustainability perspectives can lead to variations in sustainable consumption practices. The cultural dimension framework created by Refs. [92,93] has been used in several intercultural research on sustainability-related attitudes and behaviours [92]. use six cultural dimensions to identify cross-cultural differences between countries, of which power distance, collectivism, masculinity, uncertainty avoidance, and long-term orientation have been examined in sustainability-related attitudes and actions. While [93] departs from individuals’ value preferences, which are influenced by norms specific to their culture. As a result, a significant relationship was noted between four factors, i.e., conformity, self-direction, benevolence, and universalism toward sustainability. Nonetheless, both models enable the establishment of connections between culture and sustainability.

[94], have categorised and organised the benefits of the concept of cultural sustainability into seven categories: cultural heritage, cultural vitality, economic viability, cultural diversity, locality, eco-cultural resilience, and eco-cultural civilisation. This was identified as the seven “storylines” of the discourse on cultural sustainability. Furthermore, a recent study by Ref. [54] has adopted a framework of [94] on cultural sustainability and confirmed that land cover change affects cultural sustainability. The areas with high land cover change saw high cultural change, negatively affecting all seven cultural sustainability indicators. Locality negatively affected diversity and eco-cultural resilience. High diversity was observed because of chieftaincy disputes, which affected the eco-cultural civilisation of the younger generation.

Moreover, adding six principles, such as material and non-material well-being, intergenerational and intragenerational equity, maintenance of diversity, precautionary principle, maintenance of cultural systems, and recognition of independence [95], has expanded the definition of cultural sustainability. In conclusion, cultural sustainability is still a relatively new idea continually being developed. Its broad definition is linked to social, economic, and ecological sustainability [94]. However, there still exists a scope to know how various cultural sustainability indicators influence SCTD. Therefore, in this research, cultural sustainability is viewed as a reflective and sensitising concept that can assess all people's diverse values and knowledge, shaping how we live today and how we will live more sustainably. Based on this notion of sustainable cultural tourism resources, this study explores the strategies facilitating cultural sustainability indicators and how these indicators are influencing SCTD. Therefore, the researchers have developed hypotheses to explore the cultural sustainability indicators and their influence on SCTD after identifying various cultural sustainability indicators and SCTD growth.

## 3 Research methodology

### 3.1 Study site

Goa is situated on the western coast of India, bordered by the Arabian Sea to the west, Maharashtra to the north, and Karnataka to the south and east. Spanning approximately 3702 square kilometres, Goa is India's smallest state by area. However, it boasts a significant cultural and economic presence due to its rich history, thriving tourism sector, and unique blend of cultural influences. Goa's distinct cultural heritage stems from over 450 years of Portuguese colonial rule, deeply influencing its architecture, language, cuisine, and religious practices. This fusion of Indian and Western traditions has made Goa a key hub for cultural tourism, with visitors drawn to its historic churches, temples, festivals, and traditional art forms like music, dance, and crafts. Goa is home to two UNESCO World Heritage Sites, i.e., the Churches and Convents of Old Goa and the Western Ghats, crucial to its cultural identity and tourism potential.

Cultural tourism is one of the most prominent sectors in Goa, attracting millions of tourists each year. However, balancing tourism growth with preserving Goa's cultural and natural resources is a pressing concern. Studying cultural sustainability indicators in this region is essential for several reasons: Firstly, Goa's unique culture faces challenges from modernisation, commercialisation, and mass tourism, potentially eroding traditional practices and values. Sustainable cultural tourism can ensure that cultural heritage is preserved while benefiting the local community. Secondly, economically, tourism plays a crucial role in Goa, contributing 16.43 % to the state's Gross State Domestic Product (GSDP) and employing approximately 35 % of the population [96]. Goa's economic growth is driven by the strong performance of its industrial sectors, such as fishing, agriculture, tourism, and pharmaceuticals [97,98]. The Goa foreign tourist arrival in 2021–2022 was approximately 0.32 lakh [98], increasing to approximately 2.92 lakh in 2022–2023 [97,99]. Similarly, the domestic tourist arrival in 2021–2022 was 34.09 lakh [98], which has increased to approximately 76.69 lakh in 2022–2023 [97,99].

The economy has seen steady growth, with tourism being the largest segment in the services sector, drawing both domestic and international visitors. At current prices, Goa's GSDP is standing at Rs. 1,00,002.25 crore (US$ 12.87 billion) in 2023-24 and is expected to stand at Rs. 1,21,309.02 crore (US$ 14.65 billion) in 2024-25. At current prices, Goa's GSDP was Rs. 914.16 billion (US$ 11.15 billion) in 2022-23. The GSDP (in Rs.) increased at a CAGR of 9.17 % between 2016-17 and 2023-24 [96]. The economy has seen steady growth, with tourism being the largest segment in the services sector, drawing both domestic and international visitors. However, unchecked growth can strain local resources and affect community well-being. Therefore, sustainable tourism models focusing on cultural sustainability can enhance long-term economic viability while safeguarding cultural assets.

Thirdly, given its reliance on tourism and the cultural appeal of its heritage, Goa is an ideal region for exploring how cultural sustainability can be integrated into long-term tourism strategies. Preserving ICH, such as festivals, religious practices, and local art forms, is crucial for enhancing tourist satisfaction while maintaining local identity. Tourism development in Goa should balance economic growth with the sustainability of its cultural resources, which is why studying these indicators becomes essential. Therefore, studying the cultural sustainability indicators in Goa, focusing on its unique mix of heritage and tourism dependency, can provide insights into developing models that protect culture while supporting economic growth.

### 3.2 Steps of scale development

**Fig. 1:**
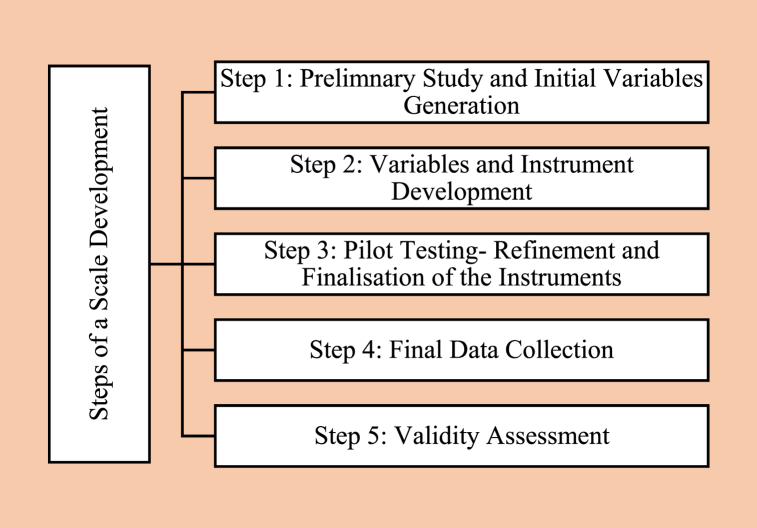
Steps of a scale development.

Source: Author's Compilation

This study aims to develop and validate comprehensive indicators of cultural sustainability and SCTD growth and how the indicators of cultural sustainability influence SCTD. Fig. 1 presents an overview of the study, which consists of five steps.

### 3.3 Step 1. preliminary study and initial variables generation

In the preliminary study, the researcher used observation to know and verify which places the tourists visit the most and to understand their modus operandi, which will help collect the data sample later. An observational study was conducted by visiting different tourist sites of Goa, such as i) Aguada Fort, which is located between the Mormugao peninsula and Calangute beach; ii) Reis Magos Fort, and Church located on the northern bank of the Mandovi River in Bardez, Goa, opposite to the capital city of Panjim; iii) The Basilica of Bom Jesus Church, located in Old Goa; iv) The religious festivals across Goa. Besides this, an observational method was also employed, complemented by conducting unstructured interviews with industry experts and academicians. Such a subjective method helped the researcher identify the most attractive places and observe their reaction when they reached a particular destination.

Further, a literature survey was conducted by identifying keywords selected based on the preliminary review of the available literature. A different set of English keywords was used, using the asterisk wildcard to include the permutation of each phrase and Boolean operators. At least two keywords were combined to enable better literature results, such as “culture and sustainability”, “sustainability and cultural indicators”, “cultural sustainability and tourism development”, and “sustainability indicators and sustainable cultural tourism development”. A Scopus database and other relevant journal websites were used to apply these keywords, which resulted in a reliable and authentic literature survey. At this stage, an attempt was made to generate variables by interviewing experts and other research scholars and asking direct and indirect qualitative questions to observe the current trend in this area of research. Thus, the observational method, literature survey, and unstructured interview helped the researcher identify the variables initially.

### 3.4 Step 2: variables and instrument development

At the initial stage, 40 variables were identified that determine the indicators of cultural sustainability and SCTD growth, and based on that, a first initial draft of a structured, closed-ended questionnaire was created. This questionnaire was then circulated among experts in the tourism field, such as academicians, PhD holders, and industry experts with more than five years of experience in the cultural and tourism industry. In total, 06 experts were approached for content validity. The experts were instructed to rate each of the 40 variables of importance and necessity based on relevance, clarity, and the simplicity of the content in each variable. The rating sheet asks the rater to indicate the following for all the scale dimensions. Here, relevance is indicated on a scale of 1–4 whether the specified variable is relevant as a measure for which it is intended. The ratings are given as 1- Not relevant, 2- Variable needs some revision, 3- Relevant but needs minor revision, and 4- Very relevant. The clarity indicated on a scale of 1–4 whether the specified variable has clarity in understanding. The ratings are given as 1- Not clear, 2- Variable needs some revision, 3- Clear but needs minor revision, and 4- Very clear. The simplicity is also indicated on a scale of 1–4, depending on whether the specified variable is simple to understand. The ratings are given as 1- Not simple, 2- Variable needs some revision, 3- Simple but needs minor revision, and 4- Very simple. The scale has been designed in such a way that it will help to test the variables and get accurate results. In addition, the experts were also asked to provide feedback on ambiguity, wording, and content about variables. Table 1 below explains the questions about measuring the cultural sustainability indicators and SCTD growth.

**Table 1:** Variables and instrument development.

Variables for Pilot Study	Variables for Final Study	Codes for Analysis	Statements	Adapted from Previous Studies/Field Experts
Var 1	Var 1	AW1	Establish awareness programs among locals and youth to care for ICH and respect local customs.	[49,72,78,100,101]
Var 2	Var 2	AW2	Better understanding of what are the sustainability and tourism expectations.	Variables identified through discussions with field experts
Var 3	Var 3	AW3	A better understanding of how tourism and culture are interrelated and their benefit to the local community.	
Var 4	Var 4	AW4	Better understanding of various strategies and how they relate to the locals.	[41,102]
Var 5	Var 5	Deleted after EFA	Funding and management solutions for tourism-related problems for the empowerment of ICH practitioners.	[78]
Var 6	Var 6	EM1	Training of community members for non-competitive tourism-related activities that complement the business.	[46,101]
Var 7	Var 7	EM2	Self-reliance of each ICH association and practitioner by having their organised system.	[46]
Var 8	Var 8	EM3	Encourage the means for local small entrepreneurs to develop and sell sustainable products that are based on the area's nature, history, or culture (including food, drink, crafts, and performance arts).	[41,78,101]
Var 9	Deleted after Pilot Study	–	Support initiatives for social and community participation and development, including, among others, infrastructure, education, health, and sanitation.	[41]
Var 10	Var 9	PD1	Parallel existence and coordination between tourism and cultural heritage management.	[41,46,49]
Var 11	Var 10	PD2	Stakeholders' initiatives and leadership in culture and tourism management.	[78]
Var 12	Var 11	PD3	Strengthen the bond of action of the companies with the conservation and enhancement of cultural heritage.	[41,46]
Var 13	Var 12	Deleted after EFA	Parallel participation in decision-making for cultural tourism sustainability.	[46,49]
Var 14	Deleted after Pilot Study	–	Stakeholders' contribution to the management of cultural resources.	Variables identified through discussions with field experts
Var 15	Deleted after Pilot Study	–	Stakeholders' contribution to ensure greater benefits to the community.	
Var 16	Var 13	Deleted after EFA	Arts and culture in education must shift from being an extra-curricular activity to being part of the core syllabus.	[41,46]
Var 17	Var 14	PM1	Establishing ICH hubs or centres and hosting tourism activities such as events, festivals, and performances.	[78]
Var 18	Var 15	PM2	Improving coordination of the various factors involved in the activity for development programs, marketing, education, and participation in the conservation and enhancement of cultural heritage.	[46]
Var 19	Var 16	PM3	Promoting and using cultural tourism to differentiate the existing tourist facility, opening new market opportunities.	[41,46]
Var 20	Var 17	Deleted after EFA	Diversify mechanisms to promote and market segments with an interest in culture.	[46]
Var 21	Deleted after Pilot Study	–	Increasing awareness and interest in ICH among locals and youth.	Variables identified through discussions with field experts
Var 22	Var 18	Deleted after EFA	Encourage participants to purchase local products and services.	
Var 23	Var 19	SP1	Allow local artists to display and perform traditional art and culture.	[103]
Var 24	Var 20	SP2	Reasonable prices can be kept for cultural products.	[78,103]
Var 25	Var 21	SP3	Facilitate the development of small and medium companies and grocery services to strengthen the system as a whole.	[49,78,103]
Var 26	Var 22	Deleted after EFA	Encourage companies to provide tourists with information on cultural activities in the destination.	[104]
Var 27	Var 23	AU1	Use appropriate and authentic cultural elements from the local region.	[45,46,49]
Var 28	Var 24	AU2	Emphasis on maintaining the original version of ICH.	[45,46,49]
Var 29	Var 25	AU3	Emphasis on maintaining/safeguarding the meaning of ICH.	[46,49]
Var 30	Var 26	AU4	Emphasis on the practitioners' identity.	[46,49]
Var 31	Var 27	CT1	Shortening presentation times of cultural activities.	[46]
Var 32	Var 28	CT2	Combining ICH with other modern performances (e.g., modern and contemporary music with traditional dances; modern materials with traditional craftsmanship).	[45,46,49]
Var 33	Var 29	CT3	Modifying lyrics or gestures to increase interaction with audiences.	[46,49]
Var 34	Var 30	CT4	Transmitting ICH as it helps understand audiences and increases interest in ICH.	[46,49]
Var 35	Var 31	Deleted after EFA	Sustainable cultural tourism can act as a well-accepted tourism product.	Variables identified through discussions with field experts
Var 36	Var 32	Deleted after EFA	Sustainable design and construction of infrastructure facilities for cultural tourists.	
Var 37	Var 33	SCTD1	Dynamic folk festivals and folklore traditions.	
Var 38	Var 34	SCTD2	Preservation of cultural resources and ruins and showcase of remarkable traditions.	
Var 39	Var 35	SCTD3	Full-time employment opportunities in the cultural tourism business.	
Var 40	Var 36	SCTD4	Community appreciation through economic and social life due to cultural tourism.	

**Note:** Four variables were deleted after the pilot study, and eight variables were deleted after EFA.

### 3.5 Step 3: pilot testing- refinement and finalisation of the instruments

Face validity was conducted through pilot testing of the questionnaire. Pilot testing was conducted to check the instrument's reliability before collecting the final data. As this study is about developing a new scale, it is important to consider the respondents at the forefront and those receiving benefits from the study. The unit of analysis for the present study is classified into two categories, i.e., cultural tourists and the residents of Goa. Cultural tourists are those who intentionally or unintentionally visit Goa's ICH resources. They are distinguished into five types: purposeful, sightseeing, serendipitous, casual, and incidental cultural tourists. They are also classified into domestic and foreign tourists. For the sample survey of tourists, a clear distinction is drawn between the types of tourists who visit the state.

On the other hand, residents are the individuals living in the study area on a full-time basis and who live at a particular place for a prolonged period (at least a year to be familiar with it); that practices, follow and show involvement in ICH resources in some or the other way. For the study, the sample of residents included officials of the Department of Tourism (DOT) Goa; Goa Tourism Development Corporation (GTDC); tour operators operating in Goa; Directorate of Art and Culture; the ICH practitioners, and locals who, in one way or another follow and practice the culture of Goa. Thus, one individual tourist and one resident are the sampling units of the study. Such sampling units above 18 years of age during the study period are the primitive respondents of this research.

The questionnaire for pilot survey results was circulated among 100 respondents consisting of 50 residents and 50 tourists from Goa. The collected data was processed through the SPSS Package (SPSS 20) to check for the reliability of the factors. The pilot survey results disclosed that the factor loading value of the variable lies within the stipulated limit of 0.50 except for 04 variables, which include Variable 09, Variable 14, Variable 15, and Variable 21. Therefore, from the expert and respondent's viewpoints, all 04 variables are discarded, and the remaining 36 variables are appropriate for the final data collection. However, expert's suggestions for revising or modifying a few variables were also incorporated.

### 3.6 Step 4: final data collection

The final data collection process was initiated in October 2022 and ended in November 2023. In the first stage, the sample was chosen based on the [105] formula, and proportionate stratified random sampling techniques were applied. This technique indicates that each stratum sample size is proportionate to the population size of the stratum when observed against the total population [106]. Here, each stratum is considered one district of Goa, i.e., North Goa and South Goa. The total population of Goa is 14, 58, 545 as per the 2011 census, of which 8, 18, 008 belong to North Goa, and 6, 40, 537 belong to South Goa.

Therefore, according to Krejcie and Morgan's sample size table, it is understood that if the total known population exceeds 2, 50, 000, a finite and standard sample can be chosen as 384. However, for the accurate result, the final sample chosen for the residents is 500, consisting of North Goa (280 residents) and South Goa (220 Residents), and tourists is 500, consisting of North Goa (367 tourists) and South Goa (133 tourists), based on the stratified sample formula below.

Thesamplesizeofthestrata=sizeoftheentiresample/populationsizexlayersize.

In the second stage, the sample distribution was divided proportionately by taluka, with 5%–10 % going to the lower population talukas and 15%–25 % to the highest population talukas. While the questionnaire was circulated, a list of cultural tourism products (ICH resources) was attached to familiarise the respondents with the research area and provide unbiased data.

The final questionnaire consists of two main sections. Section 1 deals with cultural sustainability indicators and SCTD growth variables, and section 2 deals with the respondent's demographic profile. All the 36 variables refined through the pilot survey were considered for the final data collection, which is scaled on a 7-point Likert scale. Each construct was measured using a 7-point Likert scale ranging from (1) Strongly Disagree to (7) Strongly Agree. It is a commonly used tool in social science research to capture respondents' agreement levels or disagreement with specific statements. In the end, out of 500 tourist questionnaires, 482 were found useful. On the other hand, out of 500 resident questionnaires distributed, 467 were chosen for the final study, resulting in a response rate of 96.4 % and 93.4 %. The remaining questionnaires are discarded due to missing or incomplete information.

### 3.7 Step 5: validity assessment

In the last step, the collected data was analysed using various reliability and validity statistical tools and techniques. A detailed procedure has been followed to analyse the data. Firstly, descriptive statistics, normality and multicollinearity tests were run to check whether the selected variable had any problems. Descriptive statistics is determined for each variable based on SD and mean combination. Data normalisation is tested based on skewness and Kurtosis [107]. The asymmetry skewness and kurtosis values ranging from −2 and +2 are acceptable to prove normal univariate distribution [107].

The multicollinearity test is applied based on the VIF value. It is recommended that if the value ranges from 3 to 5 VIF values, the data is free from collinearity [108,109]. However, if it exceeds the expected value, the data will be considered redundant and highly correlated to each variable in the constructs. Secondly, the dimension reduction technique under the Harman Single Factor method evaluated all the variables under a single factor to check for any bias in the data. Under this method, if the overall value of the variables is <50 %, then it is to be assumed that there is no presence of data biasedness.

Building scale typically starts with an Exploratory Factor Analysis (EFA). This method helps identify and characterise the measured variables by condensing the data from observed variables into smaller sections [110]. According to the validation requirements provided by Refs. [111,112], factor analysis ought to be carried out with the aid of EFA. In this regard, Principal Components Analysis (PCA) and varimax rotation were used to run EFA on all 36 questionnaire variables in SPSS 20.

At the same time, varimax rotation diminishes the total number of variables with high loading against each factor and further reduces smaller loadings. PCA extracts the largest variances and places them into the first factor. Using Bartlett's and the Kaiser-Meyer Olkin tests, total variance explanation and sampling adequacy were evaluated to see whether the data fit factor analysis. Further, Partial Least Square-Structural Equation Modelling (PLS-SEM) is used to examine the relationship between the constructs in Smart PLS 4.

## 4 Results and analysis

This section deals with the descriptive statistics of demographic profiles and the analysis of EFA and structural path modelling using SPSS 20 and Smart PLS 4. The detailed results are explained below.

### 4.1 Demographic profile

Table 2, based on the demographic profiles of respondents, showed that the majority, i.e., 59 %, are male compared to female, i.e., 41 %. Respondents between the age group of 31–44 years were found to be more, i.e., 36.4 %, and only 4.1 % of respondents are in the age category of 60 and above. Regarding occupation, private employees were 40.1 % of the majority, followed by 22.7 % of government employees, and only 16 % were found to be businessmen.

**Table 2:** Shows the demographic profile of the respondents (n = 949).

Attributes		Frequency (n)	Percent (%)
**Gender**	Male	560	59.0
	Female	389	41.0
**Age (In Years)**	18–30	327	34.5
	31–44	345	36.4
	45–59	238	25.1
	60 and above	39	4.1
**Occupation**	Government Employee	215	22.7
	Private Employee	381	40.1
	Business	152	16.0
	Other	201	21.2
**Marital Status**	Married	592	62.4
	Unmarried	357	37.6
**Education Qualification**	Up to 10th Grade	103	10.9
	Up to 12th Grade	204	21.5
	Graduation	304	32.0
	Post Graduation	290	30.6
	Other	48	5.1
**Annual Individual Income**	<4 lakhs (<$5, 400)	509	53.6
	Rs. 4–8 Lakh ($5, 400-$10, 700)	279	29.4
	> Rs. 8 Lakh (>$10, 700)	161	17.0

Out of the total, 62.4 % were found to be married, and the remaining 37.6 % were unmarried or single. Moreover, most of the respondents have completed and have the highest qualification as graduation, i.e., 32 %, postgraduates are 30.6 %, 12th pass are 21.5 %, and only 10.9 % have a 10th pass degree. Very few are found in the other category, i.e., 5.1 %, which includes respondents who had completed their diploma and other related courses. Regarding annual income, 53.6 % of respondents have less than Rs. 4 lakhs, and only 17 % have an annual income of more than Rs. 8 lakhs.

### 4.2 Exploratory Factor Analysis

Table 3 shows the result of EFA. The estimated chi-square value is 12449.213 with 378 degrees of freedom, indicating a significance level of 0.05. The KMO statistic of 0.881 shows that the sample adequately explains the factors. Therefore, the factor analysis conducted here can be considered a correct modus operandi to analyse the data further. After running a dimension reduction technique into SPSS 20, only 28 variables were found to have factor loading values above 0.50, and the remaining 08 were discarded due to lower factor loadings. These 08 variables include Variables 05, 12, 13, 17, 18, 22, 31, and 32. The remaining 28 variables were then factorised using PCA with the varimax rotated component matrix.

**Table 3:** Showing exploratory Factor analysis.

Factors (FA)	Variable	Codes	FA 1	FA 2	FA 3	FA 4	FA 5	FA 6	FA 7	FA 8
**Commodification and Transformation (CT)**	Var 28	CT2	0.869							
	Var 29	CT3	0.856							
	Var 27	CT1	0.803							
	Var 30	CT4	0.770							
**Authenticity (AU)**	Var 25	AU3		0.833						
	Var 26	AU4		0.822						
	Var 24	AU2		0.794						
	Var 23	AU1		0.599						
**Sustainable Cultural Tourism Development (SCTD)**	Var 34	SCTD2			0.797					
	Var 35	SCTD3			0.763					
	Var 33	SCTD1			0.722					
	Var 36	SCTD4			0.706					
**Awareness (AW)**	Var 2	AW2				0.811				
	Var 3	AW3				0.792				
	Var 1	AW1				0.699				
	Var 4	AW4				0.623				
**Empowerment (EM)**	Var 7	EM2					0.862			
	Var 6	EM1					0.727			
	Var 8	EM3					0.668			
**Parallel Development (PD)**	Var 10	PD2						0.840		
	Var 11	PD3						0.757		
	Var 9	PD1						0.658		
**Promotion (PM)**	Var 15	PM2							0.867	
	Var 14	PM1							0.697	
	Var 16	PM3							0.678	
**Sustainable Practices (SP)**	Var 20	SP2								0.835
	Var 19	SP1								0.704
	Var 21	SP3								0.683
Kaiser-Meyer-Olkin Measure of Sampling Adequacy							0.881			
Bartlett's Test of Sphericity				Approx. Chi-Square			12449.213			
				Df			378			
				Sig. value			<0.001			
Common Method Bias							30.152			
Overall Cronbach Alpha (For 28 Variables)			0.909							
Eigen Values			2.877	2.778	2.696	2.617	2.111	2.105	2.050	2.040
% of Variance Explained			10.274	9.920	9.628	9.347	7.539	7.517	7.322	7.287
Cumulative % of Variance Explained			10.274	20.194	29.822	39.169	46.708	54.225	61.547	**68.834**

The analysis yielded eight factors explaining 68.834 % of the variance. Factor 1 was labelled “**Commodification and Transformation**” due to high variable loadings. The first factor was robust, with a high Eigenvalue of 2.877, and explained 10.274 % of the variance. Factor 2 was labelled “**Authenticity**” with an Eigenvalue of 2.778, explaining 9.920 % of the variance. Factor 3 was labelled “**Sustainable Cultural Tourism Development**” due to the high loading of four variables. The variance explained by factor 3 was 9.628 % with an Eigenvalue of 2.696. Factor 4 was labelled “**Awareness**”. The variance explained by this factor was 9.347 % with an Eigenvalue of 2.617 with four variables.

Factor 5 was labelled “**Empowerment**” due to high loading, with three variables explaining around 7.539 % of the variance with an Eigenvalue of 2.111. Factor 6 was labelled “**Parallel Development**” with 3 variable loadings. This factor explains around 7.517 % of the variance with an Eigenvalue of 2.105. Factors 7 and 8 were labelled “**Promotion**” and “**Sustainable Practices**” due to high loading, with three variables in each factor. Factor 7 explains around 7.322 % of the variance with an Eigenvalue of 2.050. Whereas Factor 8 explains around 7.287 % of the variance with an Eigenvalue of 2.040.

As mentioned in the previous section, 2.3, the hypotheses are formulated based on the identified indicators and their influence on SCTD. Below are the hypotheses of the study.

H1 Authenticity has a direct influence on SCTD.

H2 Awareness has a direct influence on SCTD.

H3 Commodification and transformation has a direct influence on SCTD.

H4 Empowerment has a direct influence on SCTD.

H5 Parallel development has a direct influence on SCTD.

H6 Promotion has a direct influence on SCTD.

H7 Sustainable practices has a direct influence on SCTD.

### 4.3 Partial least square- structural equation modelling (PLS-SEM)

**Fig. 2:**
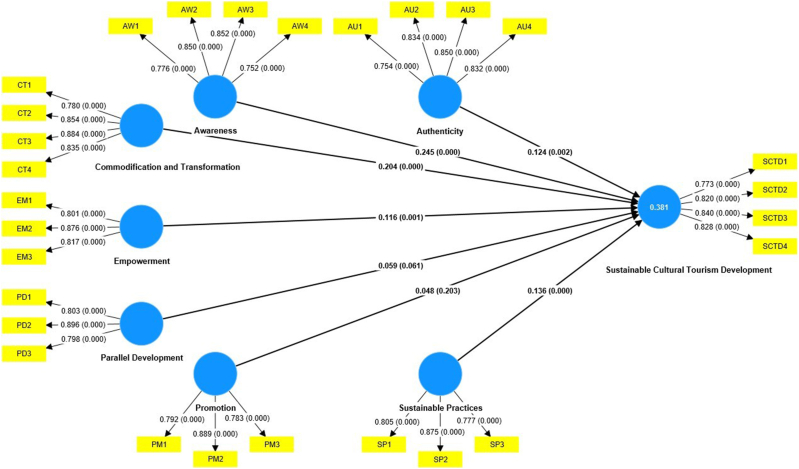
Results of PLS-SEM analysis.

This study conducted PLS-SEM analysis in two steps, including testing the reliability and validity of the measurement model and assessing the path analysis. Below is the detailed model explained (see Fig. 2).

#### 4.3.1 Measurement model

The measurement model was estimated with a bootstrap of 5000 samples in Smart PLS 4. Before constructing the measurement model, the observed variables’ mean and standard deviation scores were assessed. The highest mean value is 5.387 for (PD2), and the lowest is 4.316 for (PD3) among the constructs. The highest standard deviation value is 1.709 for (PM2), and the lowest is 1.202 for (SP3). The kurtosis and skewness values also lie within the threshold limit of −2 to +2 [107]. Hence, it proved to be acceptable for further analysis. VIF values range from 1.450 (EM2) to 2.571 (PM2), reflecting low to moderate multicollinearity. This range is considered acceptable, assuring the stakeholders of the reliability of the analysis.

Further, the dimension reduction technique under the Harman Single Factor method shows that the value is 30.152 %, confirming that they represent their corresponding factor and unbiased data.

The reliability test result is explained in Table 4, which shows that the standardised loadings of all the variables present in the study and Cronbach's Alpha (CA) of the constructs exceed the threshold limit of 0.70 [113].

**Table 4:** Shows the reliability and convergent validity of cultural sustainability indicators and SCTD.

Constructs/Variables	Standardised Loading	Standard Deviation	T Statistics	CA	CR	AVE
**Authenticity (AU)**				0.835	0.835	0.670
AU1 <- Use appropriate and authentic cultural elements from the local region.	0.754∗∗∗	0.020	37.437			
AU2 <- Emphasis on maintaining the original version of ICH.	0.834∗∗∗	0.013	64.354			
AU3 <- Emphasis on maintaining/safeguarding the meaning of ICH.	0.850∗∗∗	0.013	66.387			
AU4 <- Emphasis on the practitioners' identity	0.832∗∗∗	0.015	56.213			
**Awareness (AW)**				0.822	0.824	0.654
AW1 <- Establish awareness programs among locals and youth to care for intangible cultural heritage and respect local customs.	0.776∗∗∗	0.018	42.555			
AW2 <- Better understanding of what are the sustainability and tourism expectations.	0.850∗∗∗	0.011	74.403			
AW3 <- A better understanding of how tourism and culture are interrelated and their benefit to the local community.	0.852∗∗∗	0.011	80.234			
AW4 <- Better understanding of various strategies and how they relate to the locals.	0.752∗∗∗	0.017	45.403			
**Commodification and Transformation (CT)**				0.860	0.873	0.704
CT1 <- Shortening presentation times of cultural activities.	0.780∗∗∗	0.022	36.188			
CT2 <- Combining ICH with other modern performances (e.g., modern and contemporary music with traditional dances; modern materials with traditional craftsmanship).	0.854∗∗∗	0.013	64.766			
CT3 <- Modifying lyrics or gestures to increase interaction with audiences.	0.884∗∗∗	0.009	98.443			
CT4 <- Transmitting ICH as it helps to understand audiences and increase interest in ICH.	0.835∗∗∗	0.013	62.492			
**Empowerment (EM)**				0.776	0.775	0.601
EM1 <- Training of community members for non-competitive tourism-related activities that complement the business.	0.801∗∗∗	0.017	47.967			
EM2 <- Self-reliance of each ICH association and practitioner by having their organised system.	0.876∗∗∗	0.010	84.162			
EM3 <- Encourage the means for local small entrepreneurs to develop and sell sustainable products that are based on the area's nature, history, or culture (including food, drink, crafts, and performance arts).	0.817∗∗∗	0.015	53.172			
**Parallel Development (PD)**				0.779	0.783	0.695
PD1 <- Parallel existence and coordination between tourism and cultural heritage management.	0.803∗∗∗	0.018	44.863			
PD2 <- Stakeholders initiatives and leadership in culture and tourism management.	0.896∗∗∗	0.009	101.237			
PD3 <- Strengthen the bond of action of the companies with the conservation and enhancement of cultural heritage.	0.798∗∗∗	0.018	43.350			
**Promotion (PM)**				0.759	0.759	0.677
PM1 <- Establishing ICH hub or centres and hosting tourism activities such as events, festivals, and performances.	0.792∗∗∗	0.018	43.174			
PM2 <- Improving coordination of the various factors involved in the activity for development programs, marketing, education, and participation in the conservation and enhancement of cultural heritage.	0.889∗∗∗	0.009	95.411			
PM3 <- Promoting and using cultural tourism to differentiate the existing tourist facility, opening new market opportunities.	0.783∗∗∗	0.020	40.156			
**Sustainable Practices (SP)**				0.755	0.758	0.673
SP1 <- Allow local artists to display and perform traditional art and culture.	0.805∗∗∗	0.017	48.076			
SP2 <- Reasonable prices can be kept for the cultural products.	0.875∗∗∗	0.011	83.233			
SP3 <- Facilitate the development of small and medium companies and grocery services to strengthen the system as a whole.	0.777∗∗∗	0.019	39.914			
**Sustainable Cultural Tourism Development (SCTD)**				0.832	0.835	0.665
SCTD1 <- Dynamic folk festivals and folklore traditions.	0.773∗∗∗	0.016	46.963			
SCTD2 <- Preservation of cultural resources and ruins and a showcase of remarkable traditions.	0.820∗∗∗	0.013	64.698			
SCTD3 <- Full-time employment opportunities in the cultural tourism business.	0.840∗∗∗	0.010	82.885			
SCTD4 <- Community appreciation through economic and social life due to cultural tourism.	0.828∗∗∗	0.013	65.249			

Table 4 also estimates convergent validity results through Composite Reliability (CR) and Average Variance Extracted (AVE). The CR value ranges from 0.758 to 0.873, and the AVE score ranges from 0.601 to 0.704, which shows that the CR and AVE scores are acceptable, as they exceed the threshold limit of 0.70 and ≥0.50 [114].

The result of discriminant validity is presented in Table 5, Table 6 by applying two criteria, i.e., the Fornell-Larcker Criterion and the Heterotrait-Monotrait Ratio (HTMT). The findings of the intercorrelations suggest that the model is adequate to measure as the square root of the AVE of the constructs exceeds the corresponding constructs’ intercorrelations score [115].

**Table 5:** Shows the discriminant validity- Fornell-Larcker Criterion.

Constructs	AU	AW	CT	EM	PD	PM	SCTD	SP
**AU**	0.818							
**AW**	0.405	0.809						
**CT**	0.076	0.194	0.839					
**EM**	0.381	0.448	0.213	0.832				
**PD**	0.425	0.447	0.177	0.434	0.834			
**PM**	0.398	0.476	0.163	0.431	0.407	0.823		
**SCTD**	0.386	0.493	0.328	0.416	0.386	0.380	0.816	
**SP**	0.438	0.422	0.178	0.396	0.436	0.435	0.422	0.820

**Table 6:** Shows the Discriminant Validity- Heterotrait-Monotrait ratio (HTMT) – Matrix.

Constructs	AU	AW	CT	EM	PD	PM	SCTD	SP
**AU**								
**AW**	0.487							
**CT**	0.089	0.227						
**EM**	0.466	0.555	0.260					
**PD**	0.525	0.558	0.214	0.538				
**PM**	0.498	0.603	0.202	0.555	0.527			
**SCTD**	0.457	0.594	0.381	0.511	0.474	0.475		
**SP**	0.548	0.537	0.221	0.510	0.567	0.574	0.530	

Similarly, the maximum correlation value among the constructs was 0.603, below the most conservative critical HTMT value of 0.850. In addition, the cross-loadings of all the variables are significant at the significance level of 0.05. Hence, the study results suggested and concluded that the model offered appropriate reliability and validity evidence and, therefore, can be used to find the structural relationships among the constructs.

#### 4.3.2 Results of Path Analysis

The path coefficient of PLS-SEM in Smart-PLS 4 is also generated to check whether cultural sustainability indicators significantly influence SCTD (See Table 7). The bootstrapping test of 5000 samples is applied at the significant level of 0.05. The results were tested based on the hypotheses (H_1_-H_7_) for all the seven cultural sustainability indicators influencing SCTD. The analysis of H_1_ reveals a p-value of 0.002 and a β = 0.124, confirming a positive and significant relationship. This means that a 1 % increase in authenticity will lead to a 0.124 % increase in SCTD. Therefore, H_1_ is supported.

**Table 7:** Shows the results of the path analysis.

Hypotheses	Constructs	Standardised Estimates β	Standard Deviation	T Statistics	P Values	Inference
H_1_	AU - > SCTD	0.124	0.040	3.123	0.002	Supported
H_2_	AW - > SCTD	0.245	0.039	6.290	<0.001	Supported
H_3_	CT - > SCTD	0.204	0.029	7.148	<0.001	Supported
H_4_	EM - > SCTD	0.116	0.036	3.236	0.001	Supported
H_5_	PD - > SCTD	0.059	0.031	1.877	0.061	Unsupported
H_6_	PM - > SCTD	0.048	0.038	1.273	0.203	Unsupported
H_7_	SP - > SCTD	0.136	0.034	3.978	<0.001	Supported

The relationship between awareness and SCTD is also positively significant since the p-value is < 0.001 and the β = 0.245. This means that if there is a 1 % increase in awareness, the SCTD will increase by 0.245 %. Therefore, H_2_ is supported. Similarly, commodification and transformation positively influence SCTD, with P < 0.001 and β = 0.204, indicating that if there is a 1 % increase in commodification and transformation, there will increase SCTD by 0.204 %. Hence, H_3_ is supported. Empowerment also positively and significantly influences SCTD, with a P < 0.001 and β = 0.116. This indicates that if there is a 1 % increase in stakeholder empowerment, the SCTD will increase by 0.116 %. Thus, H_4_ is supported.

In examining the relationship between parallel development and SCTD, the statistical analysis yields a p-value of 0.061, indicating that no strong evidence exists to support the H_5_ that parallel development significantly influences SCTD. While the p-value is close to the threshold, it remains insufficient to assert a meaningful relationship. This suggests that while parallel development might intuitively contribute to SCTD by fostering collaboration, innovation, and efficiency, its influence may be constrained by factors such as coordination challenges, resource limitations, or misalignment with the unique needs of cultural tourism projects.

The analysis also indicates a p-value of 0.203 for the influence of promotion on SCTD. Since this value is considerably above the 0.05 significance level, the H_6_ is unsupported, thus indicating that promotion does not significantly influence SCTD. This result suggests that while promotional activities are critical for raising awareness and attracting tourists, they do not directly contribute to the SCTD. Furthermore, sustainable cultural practices positively and significantly influence SCTD, with a P < 0.001 and β = 0.036. This indicates that a 1 % increase in sustainable cultural practices will lead to a 0.036 % increase in SCTD occurrence. Hence, the H_7_ is supported.

## 5 Discussion

This study introduces a practically oriented indicator system tailored to Goa's unique traditions and characteristics. It presents a comprehensive framework for evaluating cultural sustainability indicators and their influence on SCTD. Through EFA, the study identifies seven primary indicators crucial to cultural sustainability AND SCTD growth. Each indicator was thoroughly explained to clarify its significance and the meaning associated with SCTD through path analysis.

**Authenticity (AU):** This indicator emphasises the practitioners' identity while utilising appropriate and authentic culture to preserve ICH's original or traditional version. This study demonstrated that authenticity is a multifaceted concept that incorporates the three criteria, i.e., the practitioners' identities, the inherited meaning, and the long-standing ICH custom [76]. The present research is consistent with [116], which upholds the relationship between authenticity and SCTD. In Goa, these three criteria can be used to determine whether an ICH was authentic in preserving the original text, preserving and protecting the meaning of the text, and emphasising the identity of the practitioners for better cultural sustainability. Crucially, these three elements interacted and converged into a sophisticated concept of authenticity rather than being mutually exclusive. To guarantee that commodified and altered versions of performances retain degrees of authenticity, practitioners who stressed the necessity of maintaining authentic ICH placed a high value on training and mastering the performance of original versions. This interpretation of ICH is influenced by objective authenticity, which gives weight to traditions and variables seen to be authentic [117].

Modernisation is progressively erasing Kampong Ayer's authentic traits and values [73]. According to Refs. [118,119], authenticity is a multifaceted concept influenced by various elements, including the location, a traditional dance performance, and visitors' and practitioners' beliefs and behaviour. That is to say, the authenticity of ICH is influenced by a practitioner's cultural identity, performances, and beliefs about audiences rather than solely dependent on one factor like gesture or spirit. According to the respondents, the transmitted performance, meaning, and message that support ICH and the practitioners' identities all impact the composite notion of ICH authenticity.

**Awareness (AW):** This indicator outlines the comprehension of cultural tourism, sustainability, and its advantages to all parties involved. In favour of this [77], stated that an “educational focus” should be placed on educating visitors about sustainable tourism and how it fits into their lives. Furthermore, a “regulatory focus” is required to establish local visitor policies and regulations, provide crime prevention teams, raise community awareness of potential criminal activity, and revise local regulations through an approval process. On the other hand [73], revealed that lack of information is a significant barrier to drawing travellers’ interest in a destination. Therefore, creating awareness about culture and its sustainability is crucial. Cultural practices remain resilient in vulnerable communities, even in multidimensional deprivation. These practices are deeply rooted in the social and territorial fabric of the community, making them critical for policies aimed at valuing both physical and symbolic local heritage.

Additionally, there are varying levels of awareness about cultural heritage sites associated with different cultural practices. This suggests that focused policies aimed at expanding and promoting cultural practices could effectively enhance the appreciation and value of the local cultural environment [103]. To address this, planners must assess stakeholders’ understanding of tourism impacts and sustainability principles before implementing participatory approaches [100]. Tourist awareness of cultural heritage also plays a role, with factors like nationality, age, and education influencing knowledge levels [120]. Developing a common understanding of sustainable tourism among stakeholders, including local communities and planners, is essential for successfully implementing sustainable development policies [121]. These findings highlight the importance of education and awareness programs for residents, tourists, and other stakeholders to promote SCTD. Such initiatives can help preserve cultural resources and ensure the long-term viability of tourism destinations.

**Commodification and Transformation (CT):** This indicator relates to the stakeholder's curiosity about whether ICH has to be changed or combined with other contemporary music to capture the audience's interest. Most respondents to this study had a favourable view regarding the commodification and change of ICH, which was an intriguing conclusion. The individuals primarily in charge of preserving the resource's authenticity are ICH practitioners [122]. Because of this, it is commonly believed that they would be against the ICH's commodification, as it could jeopardise the ICH's authenticity [123]. However, it supported the ICH's commodification and transformation to draw more tourist's attention. Shortening presentation durations, fusing ICH with other contemporary performances (e.g., modern and contemporary music with traditional dances; modern materials with conventional craftsmanship), and altering gestures or lyrics to encourage audience participation are examples of commodification and transformational changes. The primary causes of ICH practitioners' favourable sentiments regarding the commodification of ICH were their worries that audiences would find the original ICH version uninteresting and difficult to understand.

Transforming content, to some extent, boosts interest in ICH and aids in audience understanding, which makes it useful for transmission. Similarly, the original version requires more time, and the entire performance can be too difficult to play and dull for the audience. Additionally, the original ICH used simple language that all audiences could grasp because the terms were extremely strong dialectal, which the audiences could not understand. Tourism policymakers should prioritise the concept of co-creation, which enables tourists to tailor travel experiences around their specific interests, fostering more personalised and meaningful engagements. Additionally, co-creation strengthens the bond between businesses and customers by actively involving travellers in creating their experiences, enhancing customer satisfaction and loyalty [124]. This supports the collaboration between tourism and ICH developments, whereby ICH is adapted to tourists’ interests and thereby helps promote ICH as a valuable resource and draws more visitors to the area, promoting sustainable and community-based cultural tourism development.

**Empowerment (EM):** Empowerment is defined as the ability to take action, whether on an individual or collective level. While empowerment can be an effective means to enhance the capacities and resources of local communities, true community empowerment requires active participation and collective effort, rather than just individual actions [125]. In tourism, community empowerment plays a crucial role in driving sustainable tourism development [101,126]. This research emphasises the significant link between empowering communities and improving SCTD. Educating the community, empowering stakeholders to market locally-made, sustainable goods, and fostering their independence on ICH leads to empowerment. Because it allows solutions tailored to each ICH to be developed, ICH practitioners must be empowered to facilitate ICH as a sustainable tourist resource.

ICH protection and promotion are underpowered due to a common top-down development style. The research finds a positive relationship between SCTD and empowerment. Goans feel that substantial assistance from the government and other stakeholders is required to construct the ICH hub and other cultural institutions. To empower the locals, particularly the young and women of diverse religious backgrounds, the Goa Department of Culture takes the lead. The Korean government's Cultural Heritage Administration is in charge of overseeing the growth of ICH. One authority to assess and select individuals to become ICH practitioners is the head of the Cultural Heritage Administration [46].

**Parallel Development (PD):** This aspect pertains to the advantages that all parties involved stand to gain and, consequently, the growth of the tourism and cultural sectors. This research indicates that the relation between SCTD and parallel development is negligible. One possible explanation could be the divergent views of locals and other tourism sector players toward the SCTD. The respondents recommended segmenting the development of cultural tourism according to its goal (e.g., tourism or safeguarding purpose). They contended that it is critical to distinguish between the functions of pertinent ICH groups based on whether their goal is to protect, transmit, or monetise to appease tourists. For instance, the parties in charge of protecting ICH must concentrate on doing so; conversely, the parties using and promoting it must do so.

The parallel presence of tourism and cultural heritage management introduced by Ref. [127] can assist with this strategy. The supporters of the parallel existence believe that developing cultural heritage and tourism play different roles. For instance, the ownership and daily operations of the asset are within the purview of the cultural heritage development sector, while product development and marketing fall under the purview of the tourism sector. In developed destinations, parallel partnerships are typical between two sectors (such as tourism and cultural heritage development) with complementary but distinct agendas [127]. Parallel connections between cultural heritage and tourism growth can take many forms, ranging from exclusive (i.e., no communication) to symbiotic (i.e., some cooperation). Even if the relationship that respondents proposed was less clearly stated than this one, a parallel relationship approach might protect ICH's cultural values while also enabling its socio-economic value through ICH's commercial promotion.

**Promotion (PM):** This involves creating ICH hubs to market and promote cultural products to a wider audience. Encouraging people to use ICH is essential because, without locals recognising and utilising it, ICH's survival is at risk. This is especially true for the younger generation [128]. Nevertheless, the present study shows no significant relationship between SCTD and promotion. In India, Goa state is smaller than other states and has a higher proportion of rural than urban residents. Rural areas are seeing a diversity of cultures and customs. Because of this, businesses struggle to market and promote their products to a wider audience, which makes many cultural performing arts and other intangible heritages unknown to tourists. As a result of this lack of interest, knowledge, and respect for practising ICH, most respondents noted that most current generations, especially the younger generation, have less motivation to be practitioners and pose a threat to the spread of ICH.

The primary issue is that the current generation has no interest in learning about and passing along ICH [46]. It is, therefore, more challenging to embrace and transmit the value as most of this generation finds it difficult to understand [129]. noted that for the tourism business to benefit, culture and heritage must be distinctive and marketable. However, culture is never static, and when cultural symbols are required for newer, more contemporary purposes, tensions may arise that need to be addressed. When tourism does, however, there will unavoidably be a two-way interchange of ideas and cultures. In this way, visitors may see the native culture, and residents can see how the tourists represent a foreign culture.

**Sustainable Practices (SP):** This covers various methods and practices that should be followed to ensure culture's sustainability. These include allowing local artists to exhibit and perform traditional art and music, setting fair prices, and helping small and medium-sized businesses build the system. The result of this research shows that sustainable practices have a major favourable effect on SCTD, which is also consistent with the result of [130], who discussed local food, resource conservation, sustainable transportation, and recycling and waste disposal habits are some of the sustainable practices that must be followed for sustainable tourism development.

Adopting sustainability principles in everyday practices and strategic management is essential for achieving long-term goals in cultural tourism [103]. Sustainable cultural tourism protects and revitalises cultural traditions, particularly within ethnic minority communities [131]. However, mass tourism can threaten ecological sustainability and social cohesion [88]. To counter these risks, it is crucial to prioritise community participation, implement responsible policies, and incorporate local perspectives into tourism planning [131].

Furthermore, cultural heritage tourism is most effective and sustainable when integrated into a broader, diversified economic framework rather than being a community's sole source of income [88]. [132], concluded that COVID-19 has significantly influenced people's travel habits by avoiding travel in groups and deciding the destination based on their own choices, and concern about travellers' hygiene and health issues. This shift suggests that tourism stakeholders must emphasise culturally sustainable practices to maintain attractive and safe destinations, aligning with travellers' evolving preferences for cleanliness and health-conscious environments. Therefore, it is important to adopt healthy habits that will help to attract tourists and generate demand for cultural items.

## 6 Conclusion

In recent years, the tourism and hospitality sectors have benefited greatly from cultural tourism. This kind of tourism enhances the quality of life for locals, which boosts revenue for the cultural destination and encourages a more sustainable tourism model. The present study identifies the seven key indicators of cultural sustainability: authenticity, awareness, commodification and transformation, empowerment, parallel development, promotion, and sustainable practices. However, out of the seven indicators, only authenticity, awareness, commodification and transformation, empowerment, and sustainable practices were found to significantly influence the SCTD, whereas parallel development and promotion are unfavourable towards SCTD.

Cultural tourism is a significant area of research due to its relevance. Regarding the management and preservation of ICH where tourism intersects, the framework of cultural sustainability for Goa created in this paper can act as a viable tool for economic development. The Goan people's identity, authenticity, and commodification of tourism products depend on cultural sustainability. Goa has many activities through which it can preserve and promote cultural resources from various angles. For instance, host communities, especially ICH performers and local artists, can showcase traditional performing arts and music events, particularly during the busiest travel season. They should aim to draw a wider audience by staging events at popular tourist destinations, such as forts, monuments, churches, temples, and other mass tourist areas. In addition, locals can serve tourists homemade or locally grown food as another example of an ICH activity.

The results also offer useful insights for tourism enterprises looking to enhance visitor engagement and communication on sustainable practices. Furthermore, improved awareness and knowledge boost the adoption of SCTD since the present tourist generation is interested in supporting local businesses and consuming more locally, which is especially important in Goa. Moreover, the community's involvement and constant appreciation of sustainable methods and practices are also significant for enterprises and cultural destinations. Such communities are inspired to work in the cultural tourism sector and carry on the legacy in a way that will be useful and efficient for future generations. Host communities should put efforts into making tourists receive a memorable experience. On the other hand, tourists should support local communities by investing their time and money into getting authentic cultural experiences to achieve a win-win situation and the SCTD target. The state's circumstances are far better than other localities with international tourism initiatives. In line with the UNWTO's new framework for SCTD, if culturally sustainable, tourism can grow as a potent tool to support and promote the economic and social empowerment of the local population.

### 6.1 Theoretical implications

The first theoretical contribution of the study is introducing and empirically testing a new scale for measuring the indicators of cultural sustainability and SCTD growth to fill a gap in the existing literature. Due to the complexity of the terminologies and concepts, the literature review has revealed that more research needs to be done to understand cultural sustainability indicators and their influence on SCTD. Therefore, this study primarily focuses on a methodological approach in developing and validating a new scale. This scale can be used for further research and practical assessment of cultural tourism projects. Except for deleting several variables, the seven indicators of cultural sustainability are identified as the best predictor in the context of Goa.

These seven indicators are authenticity, awareness, commodification and transformation, empowerment, parallel development, promotion, and sustainable practices. Compared with the first draft of 40 variables, this study deleted 04 variables (at the face validity and content validity stage), and 08 variables were deleted after the final data collection and analysis, as the factor loading of these variables was below 0.50. Even though deleting 12 variables, the structure of seven dimensions of cultural sustainability is still confirmed, indicating good universality. The second theoretical contribution is to build and confirm the relationship model among cultural sustainability indicators and SCTD. Out of the seven indicators of cultural sustainability, only five are significant, i.e., authenticity, awareness, commodification and transformation, empowerment, and sustainable practices, which positively influence SCTD. On the other hand, parallel development and promotion are insignificant to SCTD.

### 6.2 Practical implications

The study has important practical contributions. Firstly, this study proved that authenticity, awareness, commodification and transformations, empowerment, and sustainable practices are the best predictors of SCTD. Therefore, more attention should be given to cultural innovation to the inheritance of traditional art and culture. Through cultural innovation, which will be more acceptable for travellers in the form of traditional music, dance, drama, folk art, and other entertainment, and also serves as the foundation for enhancing tourism's attraction, influence, and competitiveness. Destination managers should prioritise initiatives that preserve cultural assets' authenticity and enhance visitor and resident awareness of local cultures and heritage. This can include heritage conservation programs, cultural education initiatives, and interpretive experiences that promote meaningful interactions between visitors and local communities. Moreover, a concise and easily understood pamphlet describing Goa's culture and its significance for safeguarding the region's cultural treasures should be published by tourism groups and other interested parties. It should be helpful to both locals and visitors. A sketch map of Goa's culture, as well as details on the cultural history, tourist attractions, things to do, how to get there, and things to buy as mementoes, should all be included in this brochure.

There is a need to engage local communities in tourism decision-making processes, actively empower them to participate in tourism development initiatives and be self-reliant on each ICH association and practitioner by having their organised system. Encourage community-led initiatives prioritising sustainable practices, such as allowing local artists to display and perform their traditional art and culture, paying reasonable prices for cultural experiences, and facilitating small and medium enterprises to strengthen the cultural tourism system and sustainable livelihood opportunities. A need also arises to implement policies and regulations to manage the commodification of cultural resources and mitigate negative impacts associated with over-commercialisation and transformation. Balancing such tourism development with preserving cultural integrity and social well-being will ensure the long-term sustainability of cultural tourism destinations.

Secondly, although the current study does not support the relationship between parallel development and SCTD, parallel development is strongly connected with cultural sustainability indicators. As an integrative and collaborative model of SCTD, it will bring endless benefits to all the stakeholders (including tourists, residents, and tourism organisations such as GTDC, DOT, Directorate of Art and Culture, and other principal service providers such as hoteliers, transport, and food and beverages), in cost savings, local and national economic development, collaborations and partnerships, and diversified cultural tourism products. Such integration should be based on cultural heritage conservation and deal with two aspects, i.e., the relationship between conservation and local development and between cultural tourism/tourists and the support of local people. Therefore, this study is helpful for all the stakeholders to understand how parallel development is employed and what antecedents will influence the formation of parallel development.

For continued growth of cultural tourism, one requires more people (staff and tourists), additional cultural resources, increased funding, and a possible expansion to year-round cultural tourism offerings (most locations only operate during summer months). A clear focus on the involvement of Goan youth was emphasised to preserve culture and instil a sense of pride in younger generations. It was also recognised that concerted efforts must be made among Goan communities regarding clear communication, collaboration, and networking. Thus, connecting the two districts of Goa, sharing knowledge among these districts, and learning from each other is essential for growth.

Thirdly, further discussion on the future vision of Goa's cultural tourism acknowledged the problem of the invisibility of the Goa culture. This study fails to accept the relationship between promotion and SCTD. However, as promotion is found to be the best predictor of cultural sustainability indicators, there is still room to address this issue via improvements in marketing and promotional activities. A general increase in the quantity and quality of advertising was emphasised, as well as the need for organised marketing plans. The remoteness of certain tourist locations and the need for clear mapping and directions to such places can be addressed in addition to creating attractive brochures, using television advertising, and developing and enhancing websites. The literature emphasises the importance of proper destination marketing, stating that potential tourists base their decisions on the marketing and images they see related to aboriginal tourism. In addition, by triggering a “demonstration effect” based on face-to-face communication between hosts and guests, tourism can make a meaningful contribution to cultural sustainability and vice versa. The continued and substantial growth of the sustainable tourism subfield over a few years shows a progression toward maturity; however, there is still some distance to travel. Therefore, building long-term projects and partnerships with local communities to employ local people, involvement and improving the environment by actively exploring so much is essential for SCTD.

### 6.3 Study limitations and future research directions

There are some limitations to this research that give a researcher future direction in this area of research. First, sustainable tourism development is a broad concept that has been widely accepted, and applying this concept to research to develop any area, its adaptation must be achieved before implementation. The complexity and breadth of this framework can lead to difficulties in tailoring specific strategies for any cultural tourism destination. The lack of a universally agreed-upon definition for sustainable cultural tourism can create challenges in implementation. Future research should focus on refining and operationalising the concept of sustainable cultural tourism for specific regions or types of cultural assets. Comparative studies across different cultural destinations could explore how various aspects of sustainability can be integrated in a balanced way to suit regional contexts, particularly in developing economies or fragile cultural heritage sites.

Second, developing tourism attractions within a sustainable framework requires in-depth knowledge of the specific area, including its cultural, historical, and environmental nuances. Thus, researchers have struggled with a lack of comprehensive economic data on cultural tourism. Therefore, more case-specific studies should be conducted using participatory research methods that involve local communities in planning and decision-making. Additionally, leveraging qualitative methods such as ethnographic fieldwork and participatory mapping can provide deeper insights into the area's cultural dynamics. These approaches would help align tourism development closely with local priorities and economic values. Finally, while quantitative methodology is the best method to apply, this study still found a problem of sampling biases as the demographic profile of the sample does not align with the actual population. Future studies should also emphasise larger and more representative sample sizes, mainly including marginalised communities or groups often overlooked in tourism studies. Thus, this will address the possible problems of case-building and confirmation bias.

## CRediT authorship contribution statement

**Sadanand Gaonkar:** Writing – review & editing, Writing – original draft, Validation, Software, Methodology, Investigation, Formal analysis, Data curation, Conceptualization. **Sitaram V. Sukthankar:** Writing – review & editing, Supervision, Project administration.

## Ethical clearance

The data was anonymised and aggregated, posing no risk to individuals or communities. The data analysis does not impact any person's privacy, rights, or well-being. Ethical considerations regarding data handling and analysis were strictly followed to ensure compliance with all relevant guidelines and standards.

## Data availability statement

Data will be made available on request. For requesting data, please write to the corresponding author.

## Funding

This research received no specific grant from public, commercial, or not-for-profit funding agencies.

## Declaration of competing interest

The authors declare that they have no known competing financial interests or personal relationships that could have appeared to influence the work reported in this paper.
