# Supplementary material for: Measuring and evaluating the influence of cultural sustainability indicators on sustainable cultural tourism development: Scale development and validation
Source: Heliyon. 2025 Feb 7;11(4):e42514. doi: 10.1016/j.heliyon.2025.e42514 (PMC11872586; doi:10.1016/j.heliyon.2025.e42514)
Supplement: Multimedia component 1 [file mmc1.docx]

# QUESTIONNAIRE

Dear Sir/Madam,

I am Mr. Sadanand Gaonkar, a Research Scholar conducting research on **“Measuring and evaluating the influence of cultural sustainability indicators on sustainable cultural tourism development: Scale development and validation”.** I request you to spare some time to answer the following questions kindly. I assure you that any information furnished by you will be used only for research purposes and will be strictly kept confidential.

# Cultural Tourism Sustainability

1. The following statements are given concerning Cultural Tourism Sustainability. Kindly give your response of agreement or disagreement by tick-marking the respective column. Note: (1) Strongly Disagree, (2) Disagree, (3) Somewhat Disagree, (4) Neither agree nor disagree, (5) Somewhat Agree, (6) Agree, & (7) Strongly Agree.

| **Statements** | **1** | **2** | **3** | **4** | **5** | **6** | **7** |
| --- | --- | --- | --- | --- | --- | --- | --- |
| Establish awareness programs among locals and youth to care for ICH and respect local customs. |  |  |  |  |  |  |  |
| Better understanding of what are the sustainability and tourism expectations. |  |  |  |  |  |  |  |
| A better understanding of how tourism and culture are interrelated and their benefit to the local community. |  |  |  |  |  |  |  |
| Better understanding of various strategies and how they relate to the locals. |  |  |  |  |  |  |  |
| Funding and management solutions for tourism-related problems for the empowerment of ICH practitioners. |  |  |  |  |  |  |  |
| Training of community members for non-competitive tourism-related activities that complement the business. |  |  |  |  |  |  |  |
| Self-reliance of each ICH association and practitioner by having their organised system. |  |  |  |  |  |  |  |
| Encourage the means for local small entrepreneurs to develop and sell sustainable products that are based on the area’s nature, history, or culture (including food, drink, crafts, and performance arts). |  |  |  |  |  |  |  |
| Parallel existence and coordination between tourism and cultural heritage management. |  |  |  |  |  |  |  |
| Stakeholders’ initiatives and leadership in culture and tourism management. |  |  |  |  |  |  |  |
| Strengthen the bond of action of the companies with the conservation and enhancement of cultural heritage. |  |  |  |  |  |  |  |
| Parallel participation in decision-making for cultural tourism sustainability. |  |  |  |  |  |  |  |
| Arts and culture in education must shift from being an extra-curricular activity to being part of the core syllabus. |  |  |  |  |  |  |  |
| Establishing ICH hubs or center’s and hosting tourism activities such as events, festivals, and performances. |  |  |  |  |  |  |  |
| Improving coordination of the various factors involved in the activity for development programs, marketing, education, and participation in the conservation and enhancement of cultural heritage. |  |  |  |  |  |  |  |
| Promoting and using cultural tourism to differentiate the existing tourist facility, opening new market opportunities. |  |  |  |  |  |  |  |
| Diversify mechanisms to promote and market segments with an interest in culture. |  |  |  |  |  |  |  |
| Encourage participants to purchase local products and services. |  |  |  |  |  |  |  |
| Allow local artists to display and perform traditional art and culture. |  |  |  |  |  |  |  |
| Reasonable prices can be kept for cultural products. |  |  |  |  |  |  |  |
| Facilitate the development of small and medium companies and grocery services to strengthen the system as a whole. |  |  |  |  |  |  |  |
| Encourage companies to provide tourists with information on cultural activities in the destination. |  |  |  |  |  |  |  |
| Use appropriate and authentic cultural elements from the local region. |  |  |  |  |  |  |  |
| Emphasis on maintaining the original version of ICH |  |  |  |  |  |  |  |
| Emphasis on maintaining/safeguarding the meaning of ICH. |  |  |  |  |  |  |  |
| Emphasis on the practitioners’ identity. |  |  |  |  |  |  |  |
| Shortening presentation times of cultural activities |  |  |  |  |  |  |  |
| Combining ICH with other modern performances (e.g., modern and contemporary music with traditional dances; modern materials with traditional craftsmanship). |  |  |  |  |  |  |  |
| Modifying lyrics or gestures to increase interaction with audiences. |  |  |  |  |  |  |  |
| Transmitting ICH as it helps understand audiences and increases interest in ICH. |  |  |  |  |  |  |  |
| Sustainable cultural tourism can act as a well-accepted tourism product. |  |  |  |  |  |  |  |
| Sustainable design and construction of infrastructure facilities for cultural tourists. |  |  |  |  |  |  |  |
| Dynamic folk festivals and folklore traditions. |  |  |  |  |  |  |  |
| Preservation of cultural resources and ruins and showcase of remarkable traditions. |  |  |  |  |  |  |  |
| Full-time employment opportunities in the cultural tourism business. |  |  |  |  |  |  |  |
| Community appreciation through economic and social life due to cultural tourism. |  |  |  |  |  |  |  |

# Demographic Profile of the Respondent

| **Name:** | | | | | | |
| --- | --- | --- | --- | --- | --- | --- |
| 1 | Gender | Male | Female | | | |
| 2 | Age (In Years) | 18-30 | 31-44 | 45-59 | 60 and above | |
| 3 | Occupation | Government Employee | Private Employee | Business | Other | |
| 4 | Marital Status | Married | Unmarried | | | |
| 5 | Education Qualification | Up to 10^th^ Grade | 12^th^ Grade | Graduation | Post Graduation | Other |
| 6 | Annual Individual Income (domestic/ foreign tourist) | < Rs. 4 lakhs (Less than $5, 400) | Rs. 4-8 Lakhs ($5, 400-$10, 700) | | > Rs. 8 Lakhs (More than $10, 700) | |

**-----------------------------------------------------------THANK YOU--------------------------------------------------------**
